# Contribution of Skin Biopsy in Peripheral Neuropathies

**DOI:** 10.3390/brainsci10120989

**Published:** 2020-12-15

**Authors:** Maria Nolano, Stefano Tozza, Giuseppe Caporaso, Vincenzo Provitera

**Affiliations:** 1 Department of Neurology, Istituti Clinici Scientifici Maugeri IRCCS, 27100 Pavia, Italy; giuseppe.caporaso@icsmaugeri.it (G.C.); vincenzo.provitera@icsmaugeri.it (V.P.); 2 Department of Neurosciences, Reproductive and Odontostomatological Sciences, University “Federico II” of Naples, 80131 Naples, Italy; ste.tozza@gmail.com

**Keywords:** skin biopsy, small fiber neuropathy, autonomic neuropathy, large fiber neuropathy

## Abstract

In the last three decades the study of cutaneous innervation through 3 mm-punch-biopsy has provided an important contribution to the knowledge of small fiber somatic and autonomic neuropathies but also of large fiber neuropathies. Skin biopsy is a minimally invasive technique with the advantage, compared to sural nerve biopsy, of being suitable to be applied to any site in our body, of being repeatable over time, of allowing the identification of each population of nerve fiber through its target. In patients with symptoms and signs of small fiber neuropathy the assessment of IntraEpidermal Nerve Fiber density is the gold standard to confirm the diagnosis while the quantification of sudomotor, pilomotor, and vasomotor nerve fibers allows to evaluate and characterize the autonomic involvement. All these parameters can be re-evaluated over time to monitor the disease process and to evaluate the effectiveness of the treatments. Myelinated fibers and their receptors can also be evaluated to detect a “dying back” neuropathy early when nerve conduction study is still normal. Furthermore, the morphometry of dermal myelinated fibers has provided new insight into pathophysiological mechanisms of different types of inherited and acquired large fibers neuropathies. In genetic neuropathies skin biopsy has become a surrogate for sural nerve biopsy, no longer necessary in the diagnostic process, to study genotype–phenotype correlations.

## 1. Introduction

In the last three decades, the analysis of cutaneous nerves sampled by means of 3 mm punch biopsy has provided an important contribution to our knowledge of peripheral sensory and autonomic neuropathies [1,2].

This because the skin includes a rich network of sensory nerves originating from the dorsal root ganglia and autonomic nerves originating from the postganglionic sympathetic endings.

The skin shares with the CNS the embryologic origin from the same layer and represents, beside a physical, chemical, and immunological barrier for the entire body surface, our interface with the outside. Due to the close relationship of epidermal nerves with keratinocytes and the presence of common sensory receptors, the epidermis can be considered as a huge sensory organ able to inform our brain on what is happening outside our body and how comfortable, or not, we feel with environmental changes. This implies a continuous flow of information from the periphery to the sensory brain cortex but also to limbic areas through the insula (interoception) and then to the central autonomic network that through the cutaneous autonomic nerves puts in place emotional and homeostatic responses to outer and inner changes.

### Search Strategy

For this review, we identified the references by searching the PubMed database between January 1990 and September 2020 using the terms “peripheral neuropathy” and “skin biopsy”. The search retrieved 179 manuscripts. We did not consider single case reports or papers with populations too small to be representative. We assessed the remaining papers based on their relevance to the field and included all the literature that was appropriate for the scheme of this narrative review.

## 2. Cutaneous Sensory and Autonomic Endings

### 2.1. Intraepidermal Nerve Fibers (IENFs)

**Figure 1:**
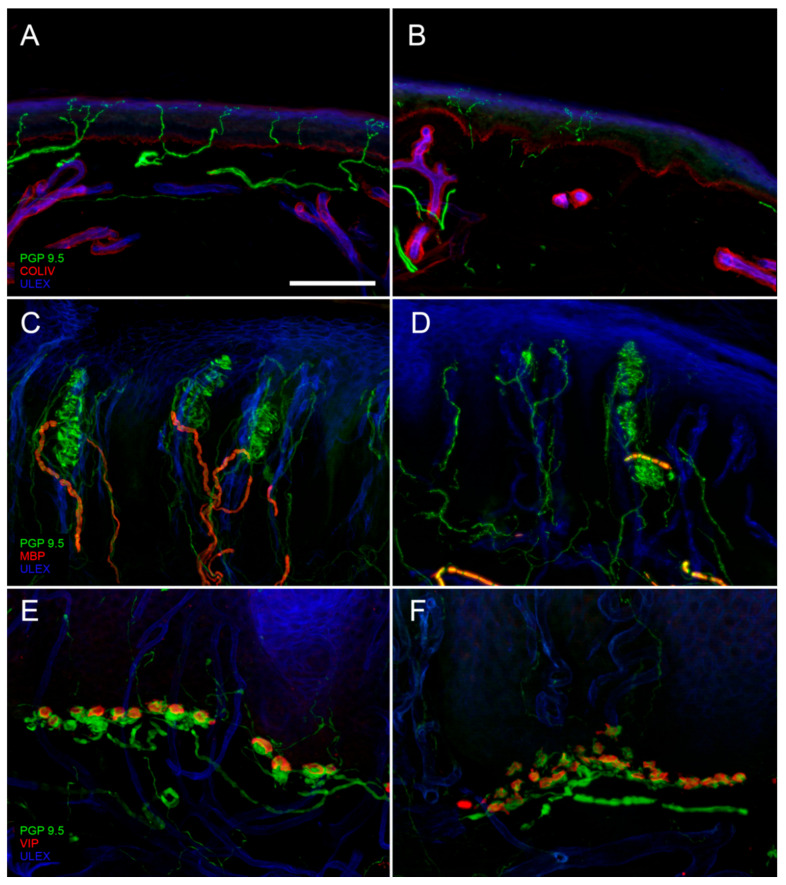
Digital confocal images from hairy (**A**,**B**) and glabrous skin (**C**–**F**) showing epidermal and dermal innervation in a healthy control (**A**,**C**,**E**) and in a patient with diabetic neuropathy (**B**,**D**,**F**). In the patient skin, a loss of Intraepidermal Nerve Fibers (IENF) is evident ((**B**) compared to (**A**)) as well as a loss of Meissner corpuscles and their myelinated afferences associated to morphological abnormalities of the surviving structures ((**D**) compared to (**C**)). Moreover, morphological abnormalities of a Merkel complex (axonal swelling and fragmentation) are present ((**F**) compared to (**E**)). In green: nerves marked with the pan-neuronal protein gene product 9.5 (PGP9.5) antibody. In blue: endothelia and epidermis marked with ULEX europaeus. In red: basement membrane and blood vessels marked with Collagen IV (Col IV) antibody in (**A**) and (**B**); myelined fibers marked with myelin basic protein (MBP) antibody in (**C**) and (**D**); Merkel cells marked with vasoactive intestinal peptide (VIP) antibody in (**E**) and (**F**). Scale bar: 100 µm in (**A**–**D**); 50 µm in (**E**,**F**).

First described by Paul Langerhans [3] and later by Arthur and Shelly [4] and Cauna [5], epidermal nerve fibers could be finally reliably and unequivocally demonstrated using the pan-neuronal marker protein gene product 9.5 (PGP 9.5) [6]. Thanks to PGP 9.5 antibodies, it has been possible to visualize in skin samples nerve bundles that from the subepidermal neural plexuses reach and penetrate the basement membrane, running with an ascending course between the keratinocytes. IENFs innervate the entire epidermis until the stratum corneum as naked axons, since they lose their Schwann cells ensheathment at the dermal–epidermal junction (Figure 1A). They are vertically oriented and may have different patterns [7]. Few of these fibers are immunoreactive (ir) to Sub P and CGRP.

IENFs are the last endings of C and A-delta fibers originating from dorsal root ganglia small neurons and mostly involved in the perception of pain and thermal stimuli. IENFs can be sub-divided into peptidergic and non-peptidergic fibers according to the expression of peptides [8]. Physiologically, peptidergic and non-peptidergic fibers terminate in different epidermal layers and have different projection patterns to the spinal cord [9].

IENFs can be found in any site of our body with higher density in proximal sites. Physiologically, the IENF density at distal leg is approximately 60% of the IENF density at thigh [10]. A reduction of IENFs density (number of fibers per linear mm) at distal leg (Figure 1B) is the hallmark to diagnose small fiber neuropathy (SFN) [11]. The advantages of applying skin biopsy compared to sural nerve analysis are the higher sensibility [12], the minimal invasiveness and then the possibility to perform it in any body site and to repeat it over time.

Two different methods of cutaneous nerves visualization are available, indirect immunofluorescence [6,13] with and without confocal analysis and bright-field immunohistochemistry [14]. For both techniques age and sex stratified normative datasets are available [10,15].

Moreover, IENFs can be analyzed using a bloodless and painless method, the skin blister [16]. Applying a capsula with a negative pressure on the skin, the epidermis separates from dermis and a blister will be obtained. The blister roof, composed only of epidermis, can be removed without damaging basement membrane and vessels. The whole sample then, formed by keratinocytes and IENFs can be processed to quantify nerve fibers with results comparable with skin biopsy [17].

### 2.2. Receptors and Myelinated Endings

In addition to free nerve endings, the skin is rich of corpusculated mechanoreceptors that are innervated by A-beta endings, provided, or not, with a capsula and able to respond with different modalities (rapidly or slowly adapting, respectively) to different kinds of stimuli. They are differently distributed in the skin and some of them are exclusively present in the hairy or glabrous skin (Meissner corpuscles, MC).

In hairy skin, hair follicle represents the main mechanoreceptor. This structure is innervated by several myelinated endings that with ascending vertical course reach the follicle, lose the myelin sheath, and envelop it constituting the characteristic “palisade endings”.

In glabrous skin, MC are uniformly distributed at the apex of dermal papillae (Figure 1C). They are provided with a capsula formed by lamellar Schwann-like cells. Their myelinated afferences, usually in a number of 1–2, reach the corpuscle, leave the myelin sheath, and undergo several branchings, with neural expansions filling the spaces inside the capsula. MCs are rapidly adapting mechanoreceptors, responsible for the high tactile acuity of the fingertip where they are mostly concentrated. The assessment of their density in the skin represents a useful measure of large fiber involvement (Figure 1D) [18].

Merkel complexes (MK) are non-capsulated, slowly adapting mechanoreceptors, distributed at the base of dermal papillae predominately in glabrous skin (Figure 1E). Their myelinated afference, in its final route, assumes a typical sigmoidal aspect. After losing its myelin sheath, it gives off several branches, ending with neural expansions with different shapes and sizes. These expansions make synapse-like contact with vasoactive intestinal peptide (VIP)-ir Merkel cells present among keratinocytes of the basal layer of the epidermis. These cells display features of sensory receptors as they are excited by mechanical stimuli and drive neural depolarization [19]. Abnormalities of the MK structure can be appreciated in case of sensory neuropathy (Figure 1F).

Pacini, Ruffini, and Krause capsula are other mechanoreceptors that are more unevenly distributed in the dermis therefore their presence in skin samples may be occasional. They are different for function and morphology, but constituted by the large expansions of neural endings originating from a large myelinated fiber after losing the myelin and branching.

Using specific markers, it is possible to study the myelinated fibers direct to the mechanoreceptors (A-beta fibers), to assess their density in the papillary dermis and their morphometry (caliber, internode and node length, and paranodal–nodal architecture). These population has a mean caliber of 3.3 µm and an internodal length around 80 µm [18,20]. This marked reduction in diameter and internodal length, compared to parental fibers in the nerve [21], may be due to a repeated branching of A-beta fibers when they reach the skin. Reduced density and morphological abnormalities of dermal myelinated fibers can be found in peripheral neuropathy (Figure 1D).

### 2.3. Autonomic Innervation

**Figure 2:**
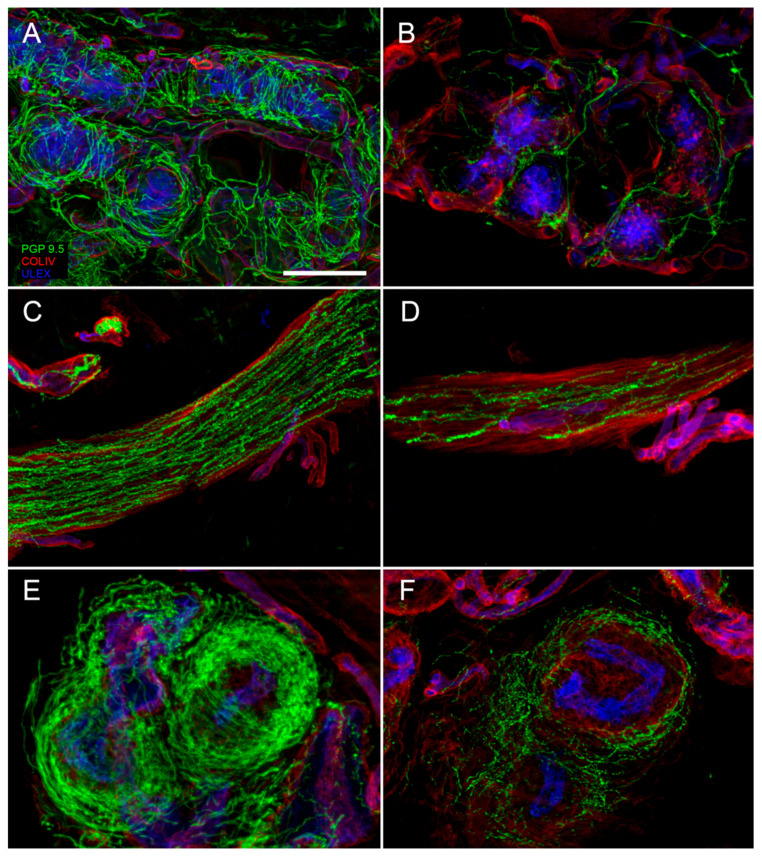
Confocal digital images showing a loss of cutaneous autonomic nerves in a diabetic neuropathy patient (**B**,**D**,**F**) compared to a healthy control (**A**,**C**,**E**). In patient skin it is evident a loss of nerves around a sweat gland ((**B**) compared to (**A**)), along an arrector pili muscle ((**D**) compared to (**C**)) and around an arterio-venous anastomosis ((**F**) compared to (**E**)). In green: nerves marked with the pan-neuronal marker, protein gene product 9.5 (PGP 9.5) antibody. In red: basal membranes and vessels marked with Collagen IV antibody. In blue: endothelia marked with ULEX europaeus (**A**–**F**). Scale bar: 100 µm.

Sudomotor nerves: Sweat glands present a dense PGP-ir network with fibers heavily encircling the sweat tubules (Figure 2A). Most of these fibers is VIP-ir, while some of them are CGRP-ir. VIP is co-expressed with cholinergic fibers and therefore it is used as surrogate cholinergic marker of sudomotor nerves. For routine purpose, sudomotor nerves can be assessed using a semiquantitative method, whereas for research different methods of quantification are available [22,23]. A loss of sudomotor nerves (Figure 2B) indicate a post-ganglionic damage of sudomotor pathway and correlates with sweating impairment.

Pilomotor nerves: Arrector pili muscle is a small band of smooth muscle fibers connecting hair follicle to the basement membrane, with mostly vestigial thermoregulation role. Pilomotor muscle is innervated by nerve fibers that go parallel to the muscle fibers direction (Figure 2C). These fibers are prevalently noradrenergic, dopamine beta hydroxylase (DβH)-ir. However, cholinergic VIP-ir fibers are also present. Semi-quantitative and quantitative methods are available to assess the pilomotor nerve fibers [24]. A reduction of pilomotor nerve density (Figure 2D) can be observed in autonomic neuropathies.

Vasomotor nerves: Arteriovenous anastomosis (AVA) is a complex structure, predominately observed in glabrous skin, constituted by vessel segments that connect small arteries with small veins. These segments are rounded by a thick muscular wall that has the role to direct the cutaneous blood flow allowing or by-passing the capillary circulation, with the main role to regulate thermo-dispersion. Heavy innervation is present surrounding the structure (Figure 2E), whereas it is less abundant around the feeding artery and sparse around the draining vein. The abundant noradrenergic (DβH-ir) innervation around the lumen, suggests its role in blood flow regulation, whereas the cholinergic innervation (VIP-ir) forms a less dense network with a possible role in vasodilation. Interesting, a further CGRP- and SubP-ir component is also present, suggesting a sensitive role as well [25]. In patients with autonomic impairment sparsely innervated AVAs can be observed (Figure 2F).

## 3. Skin Biopsy in Small Fiber Neuropathies

### 3.1. Clinical Presentation of Small Fiber Neuropathy and the Role of Skin Biopsy

In patients affected by SFN usually both sensory and autonomic components of small fibers are affected, but sensory symptoms and particularly pain, are those which more frequently lead patients to seek for medical advice.

Sensory complaints are mainly represented by spontaneous pain that usually assumes a burning connotation. However, prickling, itching, or shock-like sensation may also coexist. Evoked and uncomfortable sensations, such as hyperalgesia and allodynia, and negative symptoms, such as a reduced sensitivity to thermal and noxious mechanical stimuli, may be part of the clinical picture.

Symptoms of SFN, are usually distal symmetrical, starting at distal lower limbs and progressing in a distal to proximal fashion. This progression leads to the sequential involvement of feet, ankles, and knees before involving distal upper arms and reveals a length dependent dying-back pathological mechanism affecting the nerve fibers. Sometimes symptoms have a patchy distribution, involving proximal sites such as head, trunk, or mouth. This different distribution reveals a non-length dependent pathological mechanism due to the involvement of the sensory neuron rather than its fiber.

The diagnosis of SFN can be suspected based on the presence of characteristic symptoms and signs. According to previously published criteria [26], diagnostic confirmation can be reached by demonstrating abnormalities at quantitative sensory testing. This test, however, is by its nature a psychophysical test, and therefore dependent on the collaboration of the subject. It is recommended for screening and monitoring purposes but less suitable as a stand-alone test [27]. Quantitative sensory testing, as well as additional tests, such as laser-evoked potentials [28], contact heat evoked potentials [29], Nociceptive Flexion Reflex [30], Cutaneous Silent Period [31], ultrasonography [32], and ultrasound assisted microneurography [33] can are useful tools to study SFN [34], but, according to current guidelines, the loss of IENFs represents the morphological hallmark and the only objective test that allows to confirm a suspect of SFN.

In addition to IENF loss, some morphological changes, such as varicosity [35] and abnormalities in the distribution of nerves in the epidermis [36], may predict fibers loss. Moreover, skin biopsy can help to differentiate length dependent neuropathy from non-length dependent ganglionopathy by assessing the IENF density ratio between distal and proximal site of the lower limb [37]. This feature may guide clinicians in the SFN screening; for example, a length-dependent SFN points toward a toxic-dysmetabolic etiology, while SFN ganglionopathy suggests a dysimmune etiology.

Pain is the symptom that characterizes SFN. However, it is not possible to discriminate between patients with painful and painless SFN based on their IENF density [38].

Contradictory results have been described using other morphological features such as fibers length [39], axonal swellings [40], or the presence of GAP43-ir regenerating fibers [41]. Recently, an increase density of dermal peptidergic fibers (CGRP- and SubP-ir) has been described in patients with painful compared to painless diabetic SFN [42].

### 3.2. Causes of Small Fiber Neuropathy and the Contribution of Skin Biopsy

The commonest cause of SFN is diabetes [43]. Patients with diabetes have been among the first populations of patients in which a reduced density of IENFs was demonstrated [44]. IENF loss in diabetes is usually length dependent and is associated to a sensory dysfunction as assessed by quantitative sensory testing [45], although the loss of nerve fibers poorly correlates with the increase of sensory thresholds, regardless of the sensory modality taken into account [46]. Skin biopsy represents a useful tool to monitor the progression of the diabetic SFN. In fact, an impairment of IENFs is evident since the earliest stages of the disease. The loss of fibers correlates with the severity of the disease [47,48]. However, an SFN can manifest in patients with impaired glucose tolerance as well as in early diabetes [49].

In the last three decades, skin biopsy contributed to demonstrate small fibers involvement in an increasing number of pathologies (Table 1). In 30–50% of patients with SFN, it is not possible to identify a cause and the condition remains idiopathic [50]. In the last years, a proportion of cases of idiopathic SFN was associated with mutations in gene encoding subunits of voltage-gated sodium channels (Nav1.7, Nav.18, Nav1.9) [51,52,53]. These genetic SFN channelopathies are characterized by gain of function mutation that alters excitability of sensory fibers and causes pain. These disorders, although very rare, have provided insight into the perception of pain as well as the identification of new therapeutic targets.

Finally, growing evidence revealed a small nerve involvement in fibromyalgia, classically considered as prevalently central sensory processing disorder. A recent metanalysis showed that 49% of fibromyalgia patients presented a loss of IENFs, often described as non-length dependent [54]. This morphological evidence is further corroborated by functional studies that through microneurography demonstrated an abnormal ongoing activity of peripheral C nociceptors and increased mechanical sensitivity in fibromyalgia patients compared to controls [55]. The identification of SFN in fibromyalgia has several implications on pathogenetic, diagnostic and therapeutic aspects: (1) it shifted from central toward peripheral neuropathic pain condition; (2) it raised the question if all fibromyalgia patients need to be screened for SFN, including skin biopsy; (3) it opened the way to new potential treatments; for example, based on a possible dysimmune etiology, IVIg proved to be efficient with improvement of both symptoms and morphology [56].

### 3.3. Skin Biopsy as an Outcome Measure in Small Fiber Neuropathy

Interestingly, repeated skin biopsy over time can also be used to assess the efficacy of pharmacological and non-pharmacological treatments. In fact, a lifestyle change, including moderate physical activity and diet, showed to normalize IENF density in patients with SFN associated to impaired glucose tolerance [57]. Similarly, hormone replacement therapy has been shown to be effective for the recovery of SFN associated to hypothyroidism [58].

## 4. Skin Biopsy in Autonomic Neuropathy

Autonomic neuropathy is a disorder characterized by the selective or prevalent involvement of autonomic nerves. The onset may be acute, subacute, or chronic and from the clinical point of view it may manifest with symptoms due to the dysfunction of all domains (sudomotor, pupillomotor, gastrointestinal, genitourinary, and cardiovascular) or may be limited to only one or few of them. Abnormal pupillary reflexes, dry skin, dry mouth, dry eyes, reduced orthostatic tolerance, orthostatic hypotension, reduced gastrointestinal motility, slowing of urinary flow, and impotence may be the main signs of autonomic disfunction. The main tools available to study autonomic function are the cardiovascular reflexes with the assessment of changes in blood pressure and heart rate variability, and the cutaneous sudomotor and vasomotor function by means of Thermoregulatory Sweat test (TST), Sympathetic Skin Response (SSR), Quantitative Sudomotor Axon Reflex Test (QSART), Dynamic Sweat Test (DST), microneurography, and laser doppler flowmetry [59]. While TST [60] and SSR [61] explore the entire polysynaptic sudomotor pathway, QSART [62] and DST [63] are quantitative methods to assess postganglionic sudomotor function after pharmacological stimulation (acetylcholine or pilocarpine) by iontophoresis.

Morphological evidence of the postganglionic sudomotor damage may be obtained with the analysis of autonomic nerves in skin biopsy. This is useful since it provides information on the extent of autonomic involvement underlying autonomic dysfunction in patients with SFN. Moreover, it may allow to monitor over time such involvement through repeated biopsies.

### 4.1. Acquired Autonomic Neuropathy

Skin biopsy proved to be useful in a variety of autonomic neuropathies regardless of the etiology (Table 2) and may orientate the diagnosis by differencing between distal axonopathy (e.g., dysmetabolic) and non-length dependent ganglionopathy (e.g., autoimmune and paraneoplastic).

In the diabetic neuropathy, the more common type of autonomic neuropathy, a significant loss of sudomotor fibers [64], as well as a significant loss of pilomotor nerves [24] has been demonstrated. This cutaneous autonomic denervation is often associate to the loss of somatic small fibers, responsible respectively of anhidrosis and pain insensitivity that predispose diabetic patients to ulcers and eventually amputations.

Autoimmune Autonomic Ganglionopathy is a very rare disease due to antibody against the ganglionic receptor of acetylcholine. Patients suffer of widespread and severe involvement of both sympathetic and parasympathetic nervous system. Although the target of antibody is located on autonomic ganglia, skin biopsy in one patient demonstrated a somatic and autonomic denervation, suggesting a possible post-ganglionic neuropathy [65].

### 4.2. Hereditary Sensory-Autonomic Neuropathy (HSAN)

HSAN is a very rare disorder affecting mainly C and A-delta fibers [66]. In the last years, the original classification in five groups, proposed by Dick, continues to become more complex for the discovery of new genes and the characterization of new phenotypes. Skin biopsy proved to fill the gap originating with the availability of genetic testing that makes sural nerve biopsy unneeded for diagnostic purpose. The analysis of cutaneous innervation becomes then a surrogate of sural nerve, allowing to gain insight in the neuropathological pictures associated to the different genotypes. This has been extremely useful in some cases to understand the pathophysiology of specific autonomic disorder.

HSAN can be recessive or dominant. The recessive HSAN is usually congenital and severe disorder, due to an abnormal development of C and A-delta fibers [67]. Autonomic dysfunction is generalized and may involve multiple domains. For example, genetic mutations involving NGF-beta or its receptor (NTRK1) induce a lack of development of C and A-delta fibers that cause, respectively, congenital insensitivity to pain (CIP or HSAN V) and CIP associated to anhidrosis (CIPA or HSAN IV). Skin biopsy demonstrated in a patient with CIPA a complete lack of sensory and autonomic nerves in the skin [68] while sudomotor nerves are spared in CIP.

Moreover, CIP can be also due to biallelic mutations with loss of function in Nav 1.7 subunit [69]. Conversely, gain of function mutations in this subunit are responsible for painful conditions like familiar erythromelalgia, paroxysmal extreme pain disorder and some idiopathic painful SFN.

Insensitivity to pain with anhidrosis associated to a complete lack of cutaneous nerve fibers has been recently described in a family harboring two novel heterozygous mutations in the dystonin (DST) gene (HSAN-VI) that, unlike the previous described HSAN-VI family [70], was characterized by a non-lethal phenotype [71].

The dominant HSAN manifest with a later onset (second-third decade) and a milder phenotype. Genetic mutations cause a premature neuronal apoptosis rather than an abnormal development. Thus, phenotype is characterized by a length-dependent neuropathy with autonomic manifestation usually limited to distal anhidrosis [66].

In some autonomic neuropathies, patho-mechanism goes beyond lack of development and early neural apoptosis and is due to abnormal neuronal maturation. During development, sympathetic nerves destinated to the skin are all noradrenergic. Only after reaching their target, sudomotor nerves undergo to a noradrenergic-cholinergic shift of their phenotype. Genetic mutations in cytokine receptor-like factor 1 (CRLF1), that plays a role in this process, cause the Cold Induced Sweating Syndrome (CISS). This condition is characterized by paroxysmal sweating induced by cold and stressful stimuli limited to the upper part of the trunk, while the patients suffer of heat intolerance because anhidrotic in warm environment. Skin biopsy in a patient with CISS revealed a generalized lack of cholinergic fibers and the presence of noradrenergic fibers limited to the area of profuse paroxysmal sweating [72]. This was the first demonstration in human of the role of CRLF1 in noradrenergic–cholinergic shift of sudomotor nerves. A CISS phenotype with similar morphological findings has been described also in a patient with Stüve–Wiedemann Syndrome [73], suggesting that also the leukemia inhibitory factor receptor gene (LIFR) may play a role in postnatal cholinergic differentiation of sympathetic sudomotor neurons.

Lastly, skin biopsy in HSAN I due to mutations in *SPTCL1* gene demonstrated an increased IENFs at lower leg after treatment with l-serine [74], suggesting it to be a useful output measure.

### 4.3. Other Genetic Autonomic Neuropathies

#### 4.3.1. Hereditary Transthyretin Amyloidosis (ATTRv)

Autonomic dysfunction may be preeminent in ATTRv: early autonomic involvement of the cardiac, gastrointestinal, and genitourinary systems may manifest with symptoms that can be very disabling. It is a multi-organs disease, and it may be difficult to diagnose due to phenotypic heterogeneity. The prognosis for this severe disease has dramatically changed in the last years with the development of new disease-modifying treatments. Therefore, early diagnosis is essential for timely access to treatment. Moreover, the availability of gene silencing treatments raises important issues related to genetic screening and management of asymptomatic individuals. Skin biopsy performed in these subjects showed early loss of IENFs as well of sudomotor nerves [75]. Moreover, amyloid deposit with the Congo red were found in the dermis (especially around sweat glands) with a sensibility of 70% and a specificity of 100% in symptomatic patients, while it was present in 20% of asymptomatic subjects.

#### 4.3.2. Fabry Disease

Fabry disease is a X-linked disorder caused by mutations in the gene that codifies the lysosomal enzyme α-galactosidase A. The genetic defect causes progressive accumulation of glycosphingolipids, especially globotriaosylceramide (Gb3) in lysosomes leading to a multiorgan disease affecting the heart, kidneys, skin, eyes, central nervous system, and gastrointestinal system. The onset of symptoms occurs during childhood, with acroparesthesia, heat intolerance, gastrointestinal symptoms, and neuropathic pain. The enzyme replacement therapy points to avoid or remove Gb3 deposits; therefore, a promptly diagnosis is needed to make effective the treatment. Skin biopsy allowed to demonstrate a cutaneous denervation in 100% of the men affected patients and in 50–75% of the female and an increase of IENFs after treatment only in patients with normal renal function [76]. Moreover, a loss of sudomotor nerves in Fabry patients with sweating impairment has been demonstrated [77]. Finally using anti-Gb3 antibody, the glycosphingolipidic deposits have been found in sweat gland tubules, in arrector pili muscles and in the artery walls.

### 4.4. Idiopathic Autonomic Neuropathies

#### 4.4.1. Pure Autonomic Failure

Pure autonomic failure is a rare sporadic neurodegenerative condition, with an exclusive involvement of the autonomic system and no other neurological dysfunction. Skin biopsy provided the demonstration of a severe cutaneous denervation associated to the presence of phosphorylated alfa-synuclein deposits in autonomic nerve fibers [78], confirming the inclusion of this condition in synucleinopathies.

#### 4.4.2. Ross Syndrome

This condition was described by Ross in 1958 as being characterized by the triad abnormal sweating, areflexia, and tonic pupil. Very likely a premature apoptosis of cholinergic sudomotor neurons, leading to generalized anhidrosis and heat intolerance is at base of this disorder in which a genetic cause has not yet been demonstrated. The analysis of cutaneous innervation in 12 Ross patients revealed a complete lack of sudomotor cholinergic fibers but also a severe loss of noradrenergic and cholinergic vasomotor and pilomotor nerve fibers and a reduction of IENFs [79]. This implies that Ross syndrome is a complex disorder of the thermoregulation in which both the thermo-dispersion process (lack of sweating and vasodilation) and thermal perception are impaired.

## 5. Skin Biopsy in Large Fibers Neuropathies (LFN)

**Figure 3:**
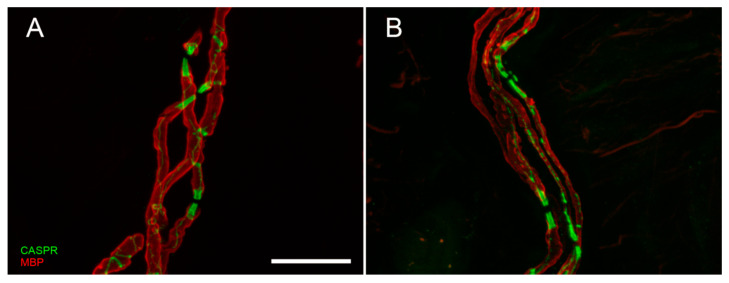
Confocal digital images of myelinated nerve fiber bundles in a control (**A**) and a diabetic patient (**B**). The double staining with anti-myelin basic protein (MBP, in red) and anti-contactin associated protein (CASPR, in green) allows to appreciate the nodal and paranodal architecture that is regular and symmetric in normal conditions, asymmetric and deranged in case of peripheral neuropathy. Scale bar: 20 µm.

Although skin biopsy represents an important tool in SFN, it provided an important contribution also in LFN. It is well-known that nerve conduction study is the first-line assessment in LFN. However, electrical stimuli do not allow to explore the very distal endings of the sensory pathway (receptors and their myelinated afferences). This distal segment, precocious involved in dying-back neuropathy, is bypassed by standard electrodiagnostic test [80]. Instead, skin biopsy allows to visualize early abnormalities of receptors and their myelinated fibers. Moreover, this approach replaces the more invasive sural nerve biopsy [81] in the study of nodal and paranodal architecture and morphometry of myelinated fibers (Figure 3A,B), widening knowledge about disease phenotypes and pathomechanisms.

Lastly, skin biopsy allowed to demonstrated small fiber involvement in LFN patients who may developed symptoms and signs of small fiber dysfunction, often overlooked when large fiber impairment is documented.

### 5.1. Charcot-Marie-Tooth Disease (CMT)

CMT is the most common hereditary neuropathy with peculiar involvement of large fiber including motor and proprioceptive nerve. However, CMT patients have signs of somatic small fibers involvement (e.g., reduced pinprick sensation) and in some CMT forms autonomic dysfunction can be clinically evident [82,83] or easily demonstrated by functional test [84].

Skin biopsy in CMT1A patients clearly demonstrated a reduced MC density, intrapapillary myelinated endings (IME) and IENF density in a length-depend fashion [84,85]. Moreover, also autonomic abnormalities (especially in the sudomotor domain) are present in patients with CMT1A [84].

Skin biopsy contributed to expand the knowledge on morphological abnormalities of nodal and internodal regions in CMT: first study showed larger nodal gaps and shorter internodal lengths in CMT1A patients without segmental demyelination suggesting a potential developmental defect during internode lengthening [85].

Another study including different CMT genotypes, demonstrated that similar nodal/internodal abnormalities were found regardless of demyelinating or axonal CMT gene [86], suggesting that mutations in both myelin and axon genes may interfere with internode development.

Lastly, skin biopsy has been proposed as outcome measure in CMT patients, although results from different studies are discrepant [84,87,88] and its role on disease progression still needs to be confirmed.

### 5.2. Other Genetic Conditions with Large Fibers Involvement

#### 5.2.1. Friedreich’s Ataxia (FRDA)

FRDA is the most frequent recessive inherited ataxia with severe involvement of large sensory nerve fibers [89]. A loss of IENFs and a reduced innervation of autonomic structures have been described in FRDA patients [90]. Interestingly, the IENF density negatively correlates with the GAA repeat expansion, supporting a genetically determined SFN in FRDA [91].

#### 5.2.2. Cerebellar Ataxia with Neuropathy and Vestibular Areflexia Syndrome (CANVAS)

CANVAS is a rare syndrome due to expansion in *RFC1* gene [92] characterized by cerebellar involvement, sensory neuronopathy and vestibular dysfunction. In about one third of patients dysautonomic dysfunction was reported with abnormal autonomic tests in half of patients [93]. Skin biopsy in two siblings revealed markedly reduced sweat gland innervation suggesting a postganglionic sudomotor dysfunction [94].

#### 5.2.3. Spino-Bulbar Muscular Atrophy (SBMA)

SBMA, popularly known as Kennedy’s disease, is a X-linked polyglutamine disorder due to degeneration of spinal and brainstem motor neurons and sensory involvement [95] with features compatible with a sensory ganglionopathy [96]. Patients with SBMA rarely report dysautonomic symptoms [97,98], but cardiovascular test showed a subclinical involvement of autonomic system [99]. Accordingly, skin biopsy from two unrelated patients showed a non-length dependent loss of IENFs, severe loss of MCs and moderate loss of autonomic innervation [100], supporting the hypothesis that SBMA may be considered as a peripheral pan-neuronopathy.

### 5.3. Inflammatory Neuropathies

#### 5.3.1. Guillian-Barré Syndrome (GBS)

GBS is an acute inflammatory neuropathy, traditionally considered to affect large myelinated fibers carrying motor functions, vibratory, and touch sensation. However, the occurrence of neuropathic pain, allodynia, reduced sensitivity to thermal or nociceptive stimuli, and autonomic dysfunctions in GBS patients raised the possibility that small fibers may also be affected. Skin biopsy from demyelinating GBS patients showed an involvement of somatic and autonomic fibers with an early loss of somatic and sudomotor nerves that remain low over time [101,102]. Intriguingly, reduced IENFs seems to be associated with disability, poor prognosis (slower recovery) and pain severity. Moreover, morphometric analysis showed segmental demyelination, axonal degeneration, swollen Schwann cells, diffuse infiltration T cell and macrophages surrounding nerves. Lastly, the study of the nodal/paranodal architecture showed a reduced sodium channels-ir, normally clustered in the node region, suggesting a spread of channels along the fibers [102].

In summary, skin biopsy demonstrated that GBS should be considered a pan-neuropathy and could be a prognostic tool.

#### 5.3.2. Chronic Inflammatory Demyelinating Polyneuropathy (CIDP)

CIDP is an acquired, immune-mediated neuropathy characterized by demyelination of nerve fibers. Although it is well-known to be a large fibers neuropathy, in some patients it is present autonomic involvement as a mild, cholinergic, and predominantly sudomotor dysfunction [103]. Skin biopsy from CIDP patients demonstrated loss of somatic small fibers and showed direct correlation between lower IENFs and autonomic symptoms [104].

Focusing on nodal/internodal architecture, skin biopsy showed shorter internodes with segmental demyelination in all patients, suggesting these features as a hallmark to distinguish CMT1A from CIDP [85]. Interestingly, the demyelinating segments were always close to the nodal regions, suggesting that paranode can represent a vulnerable region in CIDP [105].

## 6. Highlights

1. Skin biopsy is a minimally invasive technique, repeatable over time and applicable in any part of our body.
2. It allows to reach a definite diagnosis of SFN.
3. It allows to study all nerve fibers population, according to their targets and their immuno-histochemical characteristics.
4. It allows to study receptors and their afferences, morphometry, and nodal/paranodal architecture of myelinated fibers.
5. In the last decades, it allows to recognize a small fiber involvement in several hereditary and acquired conditions.
6. It is allowing to widen the clinical spectrum of peripheral neuropathy and provide new insight on the pathophysiological mechanisms.
7. It allows to monitor disease progression and to assess efficacy of pharmacological and non-pharmacological treatments.

## Figures and Tables

**Table 1:** Causes of small fiber neuropathy (SFN).

**Immuno-Mediated Disorders**	**Toxic and Drugs**
Systemic lupus erythematosus *	Antibiotics (metronidazole, nitrofurantoin, linezolid, fluoroquinolones)
Rheumatoid arthritis *	Chemotherapy (vinca, taxanes, bortezomib)
Sjögren’s syndrome *	Other drugs (flecainide, antiretroviral HIV, colchicine, statin)
Sarcoidosis	Alcohol abuse
Vasculitis *	Vitamin B6 toxicity
Inflammatory bowel disease	**Hematologic causes**
Paraneoplastic	Monoclonal gammopathy *
Celiac disease *	**Hereditary**
**Metabolic and endocrine disorders**	Nav channelopathies
Diabetes mellitus and glucose intolerance *	Familial amyloid polyneuropathy
Dysthyroidism *	Fabry’s disease
Vitamin B12 and folate deficiency *	Tangier’s disease
Dyslipidemia *	Ehlers–Danlos syndrome
Chronic kidney disease *	Familial neuropathic chronic itch
**Infections**	
HIV	
Hepatitis B	
Hepatitis C *	
Lyme disease	
Leprosy	
Systemic lupus erythematosus *	Antibiotics (metronidazole, nitrofurantoin, linezolid, fluoroquinolones)
Rheumatoid arthritis *	Chemotherapy (vinca, taxanes, bortezomib)
Sjögren’s syndrome *	Other drugs (flecainide, antiretroviral HIV, colchicine, statin)
Sarcoidosis	Alcohol abuse
Vasculitis *	Vitamin B6 toxicity
Inflammatory bowel disease	**Hematologic causes**
Paraneoplastic	Monoclonal gammopathy *
Celiac disease *	**Hereditary**
**Metabolic and endocrine disorders**	Nav channelopathies
Diabetes mellitus and glucose intolerance *	Familial amyloid polyneuropathy
Dysthyroidism *	Fabry’s disease
Vitamin B12 and folate deficiency *	Tangier’s disease
Dyslipidemia *	Ehlers–Danlos syndrome
Chronic kidney disease *	Familial neuropathic chronic itch
**Infections**	
HIV	
Hepatitis B	
Hepatitis C *	
Lyme disease	
Leprosy	

* Causes that should be included in a first level screening.

**Table 2:** Autonomic neuropathies.

**Primary**				
Autoimmune Autonomic Ganglionopathy (AAG)				
Pure Autonomic Failure (PAF)				
Ross Syndrome				
**Secondary**				
All causes of SFN (Table 1) can involve also autonomic nerve fibers				
Infectious diseases (Botulism, Diphtheria, Chagas disease)				
Lambert–Eaton myasthenic syndrome				
**Hereditary**				
**Name**	**Inheritance**	**Onset**	**Pathogenesis**	**Gene(s)**
**HSAN 1A (605712)**	Autosomal dominant	Adolescence, early adult	Reduced survival	SPTCL1
**HSAN 1B (608088)**				3p24-p22
**HSAN 1C (613640)**				SPTCL2
**HSAN 1D (613708)**				ATL1
**HSAN 1E (614116)**				DNMT1
**HSAN 1F (615632)**				ATL3
**HSAN 2A (201300)**	Autosomal recessive	Congenital, early childhood	Reduced survival	WNK1
**HSAN 2B (613115)**				FAM134B
**HSAN 2C (614213)**				KIF1A
**HSAN 2D (243000)**				SCN9A
**HSAN 3 (223900)**	Autosomal recessive	Congenital	Lack of development	IKBKAP
**HSAN 4 (256800)**	Autosomal recessive	Congenital, early childhood	Lack of development	NTRK1
**HSAN 5 (608654)**	Autosomal recessive	Early childhood to Adult	Lack of development	NGF-β
**HSAN 6 (614653)**	Autosomal recessive	Congenital	Lack of development	DST
**HSAN 7 (615548)**	Autosomal dominant	Congenital	Lack of development	SCN11A
**HSAN 8 (616488)**	Autosomal recessive	Congenital	Lack of development	PRDM12
**CISS1 (272430)**	Autosomal recessive	Congenital	Abnormal neuronal maturation	CRLF1
**TTR amyloidosis**	Autosomal dominant	Adult	Reduced survival	TTR
**Fabry’s disease**	X linked	Childhood	Reduced survival	GLA
